# Continuous Theta-Burst Stimulation Intensity Dependently Facilitates Motor-Evoked Potentials Following Focal Electrical Stimulation of the Rat Motor Cortex

**DOI:** 10.3389/fncir.2020.585624

**Published:** 2020-09-29

**Authors:** Minoru Fujiki, Yukari Kawasaki, Hirotaka Fudaba

**Affiliations:** Department of Neurosurgery, School of Medicine, Oita University, Oita, Japan

**Keywords:** corticospinal tract, electrical stimulation, motor-evoked potentials, intracortical inhibition, intracortical facilitation, theta burst stimulation

## Abstract

Although theta-burst stimulation (TBS) is known to differentially modify motor cortical excitability according to stimulus conditions in humans, whether similar effects can be seen in animals, in particular rats, remains to be defined. Given the importance of experimental rat models for humans, this study explored this stimulation paradigm in rats. Specifically, this study aimed to explore corticospinal excitability after TBS in anesthetized animals to confirm its comparability with human results. Both inhibition-facilitation configurations using paired electrical stimulation protocols and the effects of the TBS paradigm on motor-evoked potentials (MEPs) in rat descending motor pathways were assessed. Paired-stimulation MEPs showed inhibition [interstimulus interval (ISI): 3 ms] and facilitation (11 ms) patterns under medetomidine/midazolam/butorphanol (MMB) anesthesia. Furthermore, while ketamine and xylazine (K/X) anesthesia completely blocked facilitation at 11-ms ISI, inhibition at a 3-ms ISI was preserved. Continuous and intermittent TBS strongly facilitated MEPs depending on stimulus intensity, persisting for up to 25 min under both MMB and K/X anesthesia. These findings are similar to the intracortical inhibition and facilitation observed in the human motor cortex using paired-pulse magnetic stimulation, particularly the glutamate-mediated facilitation phase. However, different TBS facilitatory mechanisms occur in the rat motor cortex. These different TBS facilitatory mechanisms affect the comparability and interpretations of TBS between rat and human models.

## Introduction

The non-invasive neuromodulation method can potentially be used as an adjuvant strategy in the rehabilitation of motor and cognitive deficits caused by neurological disorders (Müller-Dahlhaus and Vlachos, 2013; Rodger and Sherrard, 2015). The effect of stimulation depends on the stimulus parameters, such as location, intensity, polarity, and frequency mode of the stimulation (Gamboa et al., 2010; Hamada et al., 2013; Nakamura et al., 2016; Shirota et al., 2017; Sasaki et al., 2018). Theta-burst stimulation (TBS) of the motor cortex (3–5 pulses at 100 Hz repeated at 5 Hz), which was originally reported in animal studies in the hippocampus of cats and rats (Hess and Donoghue, 1996), has been successfully translated in the awake human motor cortex as either intermittent and facilitatory or continuous and inhibitory TBS paradigms for motor-evoked potentials (MEPs) with repetitive transcranial magnetic stimulation (rTMS; Huang et al., 2005). Comparability, i.e., whether similar effects would be seen particularly in the descending motor system of rats, and underlying functional validations are yet to be determined. With the widespread application of TBS as a tool to modify the excitability of the human motor cortex, the present study explored corticospinal excitability after TBS using two different standard anesthetics on freely behaving animals to replicate human findings. Recent TMS-TBS protocols and MEP recording methods in animal models have been useful for translation purposes and for understanding the mechanisms underlying human results (Vahabzadeh-Hagh et al., 2011; Hsieh et al., 2012, 2015; Sykes et al., 2016). In contrast, a single pulse stimulation-MEP, such as TMS-MEP, causes activation of both the motor cortex and subcortical structures (Mishra et al., 2017); thus, focal short-burst triple-pulses for MEP have been proposed in rat models (Carmel et al., 2010; Mishra et al., 2017). Electrical motor cortical stimulation would enable focal stimulation protocols with greater specificity and accuracy for basic MEP recording, intracortical inhibition-facilitation exploration, and TBS modulation in rat models.

Therefore, we focused on electrically-induced MEPs, continuous TBS (cTBS), and intermittent TBS (iTBS), as previous work has predominantly employed TMS-MEP and TMS-TBS (Hsieh et al., 2015; Sykes et al., 2016) in rats based on the original human paradigm, but few reports have employed more focal and stable motor cortical electrical stimulation (Barry et al., 2014). This study aimed to establish an animal model that allowed for the study of factors possibly affecting MEP amplitudes, and thus cortical excitability, under a more standardized condition and with additional focal stimulation than that achieved with conventional TMS. Configuration of the induced current flow (polarity, location, and monophasic or biphasic) *via* epidural electrodes was preliminarily tested to assess whether it is compatible with TMS-induced electric fields relative to monopolar direct electrical stimulation of the motor cortex. This is important, as determining the stability of the protocol under anesthesia is required before future repeated experiments can be conducted, e.g., exploring the effects on the central nervous system (CNS) of drugs, wakefulness, and free-moving conditions. Indeed, drug effects in human results (Kujirai et al., 1993; Rothwell, 1997) and acute changes in TMS measures of motor excitability after a single-dose application (Ziemann et al., 2015) require confirmation under experimental settings.

The present results provide animal platforms in conditioned laboratory settings for pharmacological and various pathophysiological evaluations, as well as an understanding of previous human results.

## Materials and Methods

### Animals

**Figure 1:**
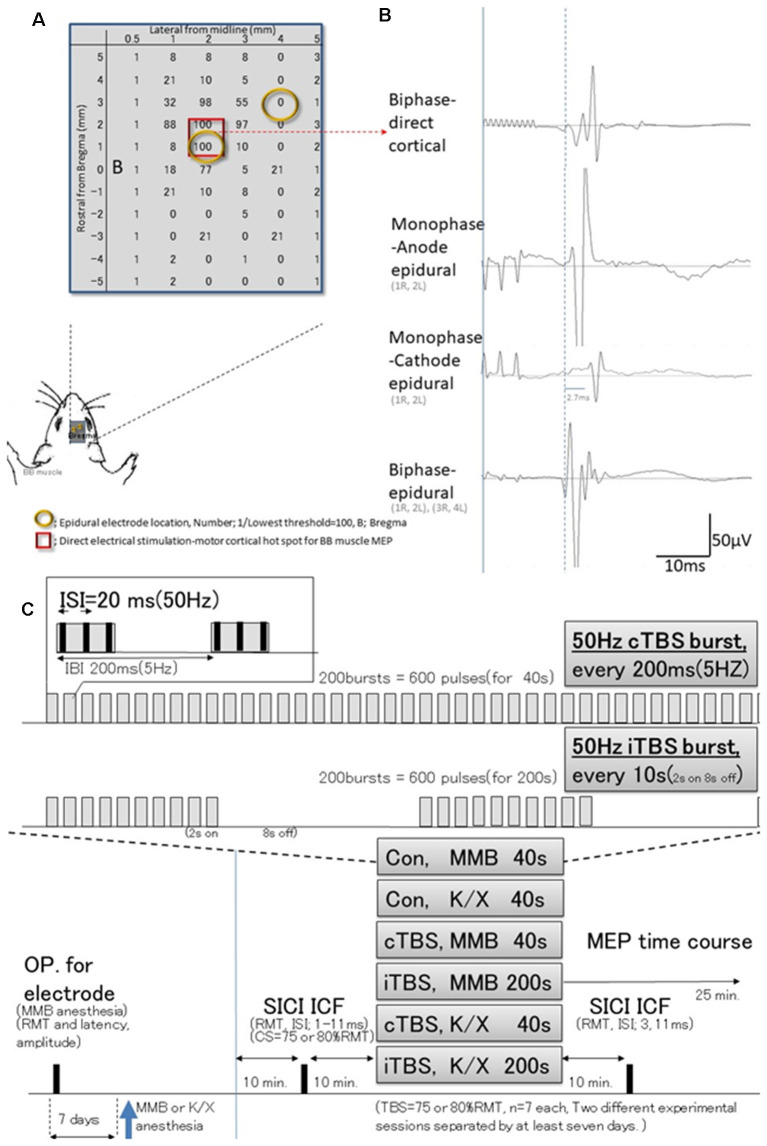
Schematic illustration of experiments; stimulus-accuracy validation and timeline; comparisons between direct motor cortical electrical stimulation induced-motor-evoked potentials (MEPs) and epidural stimulation MEPs (different configuration of the induced current flow; polarity, location, and monophasic or biphasic). **(A)** The results of cortical motor mapping (2 mm anterior and 2–3 mm lateral to the bregma) were following previous work comparing intracortical electrical stimulation and epidural stimulation (Takemi et al., 2017). Hot spot mapping for the biceps brachii muscle was identified; locations were defined as [1/(lowest threshold (mA) of direct motor cortical stimulation induced-MEPs)] 100 (red square identifies two locations). Before a full craniotomy, the location of two epidural electrodes placed over the rat’s motor cortex was systematically changed to identify the best area for eliciting MEPs *via* the motor cortex. **(B)** MEP latencies after direct motor cortical electrical stimulation corresponded with those after monophase-anode (1R, 2L) epidural stimulation and biphase-epidural stimulation [(1R, 2L) and (3R, 4L)], whereas MEPs after monophase-cathode (1R, 2L) epidural stimulation exhibited a 2.7 ms delay in latency, and lower amplitudes than other modalities. Thus, the biphasic epidural stimulation electrode was located on the motor cortical hot spot for BB muscle, inducing a horizontally oriented electric field across forelimb representation confirmed compatibility those with MEPs identified by direct cortical electrical stimulation. **(C)** Epidural electrodes were placed 7 days before testing under MMB anesthesia. The SICI and ICF were evaluated after 10 min. Base recording, cTBS, iTBS, and no-theta-burst stimulation (TBS)- were performed under MMB or K/X anesthesia at 10 min. Base recording (*n* = 7, each group). Full ISIs, as well as ISIs of 3 and 11 ms, were tested 10 min after TBS. MMB, medetomidine/midazolam/butorphanol anesthesia; K/X, ketamine and xylazine anesthesia; SICI, short-latency intracortical inhibition; ICF, intracortical facilitation; cTBS, iTBS; continuous or intermittent theta burst stimulation; ISI, inter-stimulus interval; IBI, inter-burst interval; RMT, resting motor threshold.

All experimental protocols were approved by the Ethical Committee of the School of Medicine, Oita University (protocol number 192301). Experiments were conducted on 48 adult male Sprague–Dawley rats (body weight, 290–375 g; purchased from Charles River Laboratories, Japan) housed at controlled room temperature (24 ± 1°C) with a 12/12 h light/dark cycle. The room was maintained at 24°C with constant humidity. Rat food pellets and tap water were provided *ad libitum* between experimental procedures. This study constituted six separate experimental conditions involving 48 animals [time course for no TBS (*n* = 7), cTBS (*n* = 7) and iTBS (*n* = 7) under medetomidine/midazolam/butorphanol (MMB) anesthesia; same procedures under ketamine and xylazine (K/X) anesthesia (*n* = 21); and preliminary studies (*n* = 6); see Figure 1 for details]. We used “one animal” for “two sessions” for animals used twice (see also “Paired Motor Cortex Electrical Stimulation: SICI and ICF” section for detail).

Preliminary studies were undertaken to test three different configurations of the induced current flow (i.e., polarity, location, and monophasic or biphasic) *via* epidural electrodes delivering electrical pulses at 1.2 times the resting motor threshold (RMT) of the MEPs (for which a separate set of six rats were prepared). For this study, rats (*n* = 3) were anesthetized and placed in a stereotactic frame (Figure 1). Recording methods were similar to those described elsewhere (Hsieh et al., 2012; Sykes et al., 2016). For comparison, additional rats (*n* = 3) were prepared similarly and received direct electrical stimulation of the motor cortex. Briefly, a craniectomy of 9 × 5 mm^2^, i.e., drilling above the forelimb and hindlimb regions of the sensorimotor cortex (coordinates relative to bregma: 4.5 mm caudal, 4.5 mm rostral, and 0.5–5.5 mm lateral) to expose the to-be-stimulated cortex, was performed over the motor cortex where single electrodes were positioned at different locations. Electrical stimulation consisted of 3–10 500-μs biphasic pulses (cathode first) delivered at 500 Hz, and the maximum stimulator output (MSO) was adjusted to 1.0 mA; 808 ± 33 Ω impedance. Such stimulation yielded MEPs from the forelimb biceps brachii (BB) muscle when the motor cortex was stimulated 2 mm anterior and 2–3 mm lateral to the bregma (Takemi et al., 2017). A set of two epidural electrodes placed over the rat’s motor cortex can be systematically adjusted to the best position for eliciting MEPs *via* the motor cortex (Fujiki et al., 2020). Epidural stimulating configurations were determined based on the comparison of these procedures.

### Motor Cortex Stimulation and Recording of Motor-Evoked Potentials

**Figure 2:**
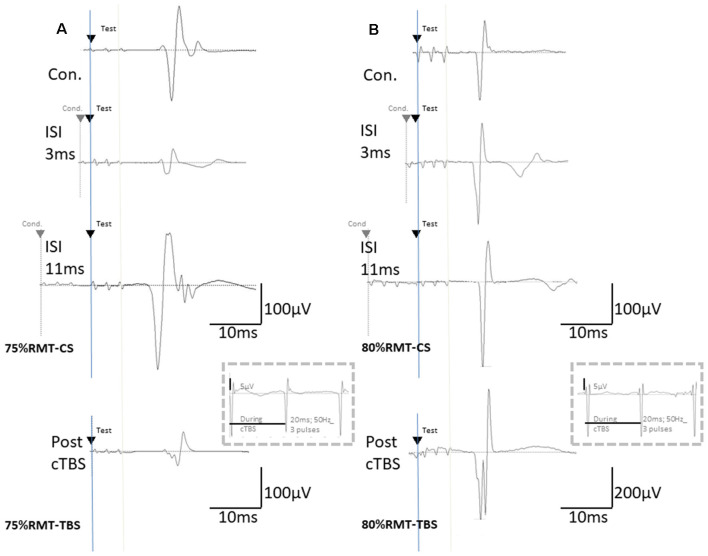
The basic waveform of MEPs recorded from the biceps is composed of short-latency (approximately 14 ms) biphasic waves. **(A)** Inhibition and facilitation of MEPs at ISIs of 1, 3, and 11 ms under MMB anesthesia at 75% RMT-CS. This phenomenon is reminiscent of the short-latency intracortical inhibition (SICI) and ICF in the human motor cortex observed when using paired-pulse TMS. MEPs at pre- and post-cTBS (top and bottom, respectively), at ISIs of 3 (second) and 11 (third) ms, and EMG recordings during cTBS (fourth trace). **(B)** Inhibition and facilitation of MEPs at ISIs of 1, 3, and 11 ms under MMB anesthesia at 80% RMT-CS. MEPs at pre- and post-cTBS (top and bottom, respectively), at ISIs of 3 (second) and 11 (third) ms, and post-cTBS (bottom), and EMG recordings during cTBS (fourth trace). Note that recordings during cTBS (fourth trace, inside dashed line boxes) show small electrical stimulation artifacts (50 Hz), three 20-ms pulses, and an absence of evoked MEPs during cTBS (**A,B**; 5 μV of amplitude calibration in the fourth trace, inside dashed line-boxes). MEPs were inhibited after cortical motor cTBS at 75% of the RMT, while they were strongly facilitated immediately after cortical motor cTBS at 80% RMT (Note: 200 μV of amplitude calibration in the bottom trace, right **B**).

The basic procedures of electrical stimulation and MEP recordings were based on methods previously described by Mishra et al. (2017). Briefly, epidural cortical stimulating electrodes [Plastics One with 1.19 mm diameter with a flat tip on two locations: 1.0 mm rostral and 2.0 mm lateral (1R, 2L) and 3.0 mm rostral and 4.0 mm lateral from bregma (3R, 4L); orange circles in Figure 1B] were placed 7 days before testing under MMB anesthesia. The screw electrodes were attached in advance to a head connector (Plastics One) such that they were secured with skull screws and dental acryl for repeated measurements (Mishra et al., 2017). To assay the descending motor systems, we stimulated the motor cortex and measured MEPs from the contralateral BB muscle. For motor cortex stimulation, a train of three biphasic square wave pulses was delivered with an isolated pulse stimulator (A-M Systems, Model 2100, Sequim, WA, USA) to achieve temporal summation for selective activation of the motor cortex (0.2 ms per pulse for each polarity; interstimulus interval of 3 ms; Figure 2A). Also, we compared latencies with three pulses to those with single pulses delivered over the motor cortex. For testing, trains of stimuli were delivered every 5 s to allow for the recovery of responses (Carmel et al., 2010).

### Paired Motor Cortex Electrical Stimulation: SICI and ICF

The RMT was determined by first decreasing the stimulator output by 0.1 mA until MEPs disappeared and then increasing the output in 0.1-mA increments until six MEPs of 50 μV (peak-to-peak) were elicited out of every 12 trains of 3-ms interval three biphasic square wave pulses. We recorded 20 min of baseline MEPs every 5 s (0.2 Hz) at 120% of RMT. Two isolated electrical stimulators connected to a single stimulus electrode with custom-made switching systems were used. Parameters were controlled for appropriate stimulus intervals and intensity like that for paired TMS (Kujirai et al., 1993; Vahabzadeh-Hagh et al., 2011; Hsieh et al., 2012). Intracortical inhibitions or facilitations [corresponding to short-latency intracortical inhibition (SICI) and intracortical facilitation (ICF) in human motor cortex using paired-pulse TMS] were tested using a paired electrical and subthreshold conditioning stimulus (CS) preceding a suprathreshold test stimulus (TS; Kujirai et al., 1993; Rothwell, 1997). Subthreshold CS was set at 70, 75, and 80% RMT, while the intensity of TS was adjusted to evoke an MEP of approximately 300 μV (peak-to-peak) in the left BB muscle. Interstimulus intervals (ISIs) of 1, 2, 3, 5, 7, 11, 13, and 15 ms were utilized to test intracortical inhibitions or facilitation. Full ISIs were tested 10 min before TBS, while ISIs of 3 ms and 11 ms were tested 10 min after TBS. Two different experimental sessions separated by at least 7 days were conducted, while one of two TBS intensity protocols were used in each session in a pseudo-randomized order.

### Motor Cortex Electrical Stimulation: cTBS and iTBS

Either a continuous or intermittent TBS (cTBS or iTBS under MMB or K/X; *n* = 7 each) or an absent (no-) TBS- (MMB or K/X; *n* = 7 each) was applied for a total duration of 40 s or 200 s, respectively. cTBS consists of a burst of three pulses at 50 Hz, repeated at 5 Hz, and delivered for 40 s continuously (600 pulses). In contrast, iTBS involves the same burst, delivered for 2 s with an 8 s off-period, consisting of 600 pulses based on original reports of rTMS of the human motor cortex by Huang et al. (2005). At the end of the data collection, rats were sacrificed humanely by an anesthetic overdose (350 mg/kg pentobarbital sodium, Henry Schein) before decapitation. Finally, extracted brains were fixed in paraformaldehyde and sectioned for the histological verification of electrode positioning.

TBS was delivered at 75 and 80% of the RMT [approximately 0.5–1.2 mA, corresponding to previous reports (Yang et al., 2019)] for 600 pulses. Also, a final 25-min MEPs post-stimulation was recorded at 0.2 Hz at 120% RMT. No-TBS- was instead delivered by unplugging the electrodes at the stimulator while the cTBS or iTBS protocol was conducted.

### MEP Acquisition

MEPs were measured *via* a stainless-steel braided wire (Cooner Wire, catalog number AS 634, Chatsworth, CA, USA) inserted into the left BB muscle. Successively, they were pre-amplified and stored (Neuropack 8, Nihon-Kohden Co. Limited, Tokyo, Japan and Brain Vision Recorder, Brain Products, Germany, with 5–3,000 Hz bandpass at a sampling rate of 5,000 Hz and 100-ms analysis time). We acquired the first 100 ms of electromyography (EMG) data after the stimulation for quantification. The EMG response diminished to baseline within this period after the presentation of the conditioning stimulus followed by a TS (see below). For motor threshold determination, recordings were obtained at regular intervals from a low cortical stimulus intensity that did not produce any motor response (subthreshold: 0.5 mA) to high intensity (3.0 mA) that saturated the MEPs.

Rats were deeply anesthetized with either a combination of MMB anesthesia (0.15/2.0/5.0 mg/kg, respectively; intraperitoneally) or a combination of ketamine (90 mg/kg) and xylazine (10 mg/kg), which was used to preserve motor responses. Anesthesia depth was monitored periodically using the pedal withdrawal (“toe-pinch”) reflex at the same relative timing and frequency in all animals. The absence of such reflex indicated that a standardized depth of anesthesia and analgesia was achieved, and this was maintained throughout electrode implantation and recording. We used a temperature-controlled heating pad to maintain the body temperature at 37°C intraoperatively both during the post-surgical recovery and the recording period. Rats were placed in a grounded stereotaxic frame (Narishige, Japan) and electrically isolated from metal ear bars using parafilm.

### Data Analysis

All MEP data were analyzed offline using Brain Vision Analyzer2 (Brain Products, Germany), as also reported by Sykes et al. (2016). Peak-to-peak MEP amplitudes were measured (at 120% RMT intensity, composed of 12 individual sweeps in each minute run). Successively, normalized amplitudes to the final 5 min of baseline amplitude were expressed as a percentage change, allowing for between-subject comparisons, and were grouped into 2-min bins and a final 3-min bin.

All data are presented as mean ± standard error of the mean (SEM). Different groups of animals were compared using a one-way (two-way for time course) analysis of variance (ANOVA) with a Student-Newman–Keul *post hoc* analysis (SPSS, Cary, NC, USA). Experiments with three or more groups were analyzed with a two-way ANOVA followed by a *post hoc* Bonferroni-Dunn test. For TBS effects, the statistical significance of group differences was analyzed with an ANOVA with time (TIME) as a within-subject factor and group (GROUP) as a between-subjects factor. This was followed by a *post hoc* Holm test. To investigate whether the time effect differed among groups, we confirmed the TIME × GROUP interaction. Differences were considered significant at *P* ≤ 0.05.

## Results

### MEP Basic Waveforms

Direct motor cortical and epidural stimulation-induced MEPs were compared for accuracy verification (different methodological configurations of the induced current flow; polarity, location, and monophasic or biphasic, see Figure 1 for detail). Quantitative differences in the final 5 min of MEP baseline parameters between the two anesthetic conditions were not observed (RMT: 1.04 ± 0.03 vs. 1.03 ± 0.03 mA; latency: 13.9 ± 0.29 vs. 13.6 ± 0.25 ms; amplitude: 286 ± 7.4 vs. 302 ± 14.8 μV; under MMB and K/X anesthesia, respectively). In addition, an absence of statistically significant effects of anesthetic combinations on RMT (*t*_(40)_ = 0.17; *P* > 0.05), latency (*t*_(40)_ = 0.94; *P* > 0.05), or amplitude (*t*_(40)_ = 0.96; *P* > 0.05) was found.

Similarly, statistically significant effects of previous TBS sessions on RMT (*t*_(40)_ = 1.56; *P* > 0.05, *t*_(40)_ = 1.59; *P* > 0.05), latency (*t*_(40)_ = 0.15; *P* > 0.05, *t*_(40)_ = 0.2; *P* > 0.05), or amplitude (t *=*_(40)_ = 0.24; *P* > 0.05, *t*_(40)_ = 1.38; *P* > 0.05, MMB and K/X anesthesia respectively) were not seen.

Following previous methodological standards (Mishra et al., 2017), motor cortical electrical stimulation elicited a clear short-latency MEP (14.1 ms in latency, not including waveforms with latencies <5 ms), as illustrated in Figure 1B. We analyzed electrophysiological changes in MEPs based on the effects of anesthetic combinations, GABA-A agonist midazolam-based MMB, and non-specific NMDA receptor blocker, ketamine-based K/X, with or without cTBS or iTBS.

### Inhibition and Facilitation Patterns of Paired-Stimulation MEPs

**Figure 3:**
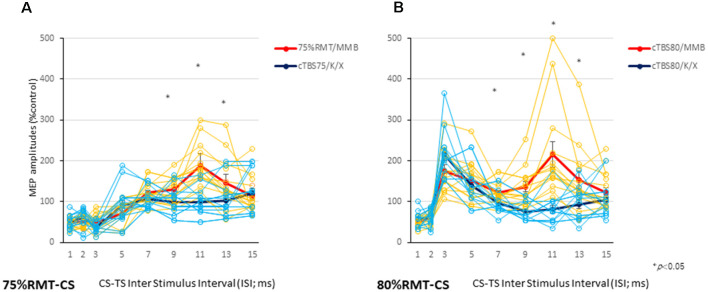
Individual and averaged MEPs in each ISI under the MMB and K/X anesthesia conditions. Individual profiles of normalized MEPs in each ISI under the MMB and K/X anesthesia conditions **(A,B)**. Averaged normalized MEPs under the MMB (red) and K/X (blue) anesthesia conditions at a CS at 75 (left **A**) and 80 (right **B**) % of the RMT preceding test stimulus (TS)-MEPs. MMB-anesthetized rats demonstrated significant inhibition at ISIs of 3 ms and facilitation at 11 ms, whereas K/X-anesthetized rats showed significant suppression at 11 ms and facilitation at 75% of the RMT-CS. Inhibition was preserved at an ISI of 3 ms (*P < 0.05). MMB-anesthetized rats presented significant facilitation at ISIs of 3 and 11 ms following inhibition at 1 ms. In contrast, K/X-anesthetized rats showed significant suppression at 11 ms and facilitation at 80% RMT-CS. An analysis of variance (ANOVA) revealed that the values were significantly greater in the MMB group than in the K/X group at an ISI of 11 ms (*P < 0.05).

CS at 75% of the RMT preceding TS-MEPs showed inhibition (ISI, 3 ms) and facilitation (ISI, 11 ms) patterns under MMB anesthesia (Figures 2A, 3A). While K/X anesthesia completely blocked facilitation at an ISI of 11 ms, inhibition at an ISI of 3 ms was preserved (*P* < 0.05; Figure 3A). A one-way ANOVA revealed a significant difference at an ISI of 9 (*F*_(1,26)_: 5.52, *P* < 0.05), 11 (*F*_(1,26)_: 26.89, *P* < 0.001) and 13 (*F*_(1,26)_: 4.41, *P* < 0.05) ms, respectively.

CS at 80% of the RMT preceding TS-MEPs showed inhibition (ISI, 1 ms) and facilitation (ISI, 3 ms, and 11 ms) patterns under MMB anesthesia (Figures 2B, 3B). While K/X anesthesia completely blocked facilitation at an ISI of 11 ms, inhibition at an ISI of 1 ms and facilitation at an ISI of 3 ms were preserved (*P* < 0.05; Figure 3B). A one-way ANOVA revealed a significant difference at an ISI of 7 (*F*_(1,26)_: 5.23, *P* < 0.05), 9 (*F*_(1,26)_: 9.72, *P* < 0.0005), 11 (*F*_(1,26)_: 19.2, *P* < 0.0005) and 13 (*F*_(1,26)_: 6.01. *P* < 0.05) ms, respectively. Therefore, the intracortical inhibition and facilitation profiles of a CS intensity at 75% of the RMT are reminiscent of the SICI and ICF in the human motor cortex using paired-pulse TMS. In contrast, such profiles of a CS intensity at 80% of the RMT were not entirely comparable to those seen in the human motor cortex using paired-pulse TMS.

Overall, given that CS at 70% of the RMT preceding the TS-MEPs revealed non-identical patterns under both conditions of anesthesia (data not shown), we chose CS intensities of 75 and 80% for the TBS procedures in the present study.

### TBS Effects on Rat MEPs

**Figure 4:**
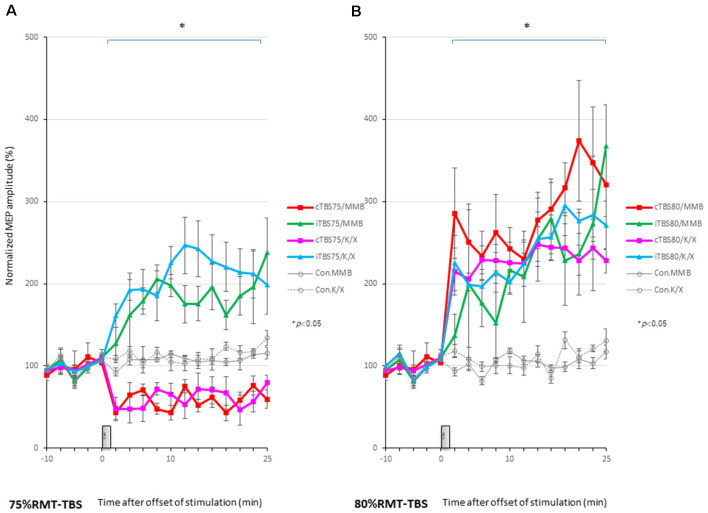
MEP amplitudes increase in intensity in the stimulated cortex after 80% RMT-cTBS and iTBS time-series data of MEP amplitudes are expressed as the percentage change from baseline attributable to TBS. Group data are presented as the mean ± standard error of the mean. **(A)** Compared to the no-TBS group, the increase in MEP amplitudes is significantly lower in the 75% RMT-cTBS group and significantly higher in the 75%-RMT-iTBS group. **(B)** The increase in MEP amplitudes in the 80% RMT-cTBS and iTBS groups is significantly greater than that in the no-TBS group. *P < 0.05.

MEPs were inhibited after motor cortical cTBS at 75% of the RMT, lasting up to 25 min under both MMB and K/X anesthesia (*P* < 0.05; Figure 2A, bottom trace, and Figure 4A red, pink line). In contrast, iTBS at 75% of the RMT facilitated MEPs, lasting up to 25 min under both MMB and K/X anesthesia (*P* < 0.05; Figure 4A green and blue line). Finally, both cTBS and iTBS at 75% of the RMT lead to MEP inhibition, while facilitation profiles were identical to those obtained using biphasic TMS at 80% of the active motor threshold (AMT) of the human motor cortex.

An ANOVA revealed a significant main effect of group on MEP, whereby the effects of the stimulation differed among the six groups (main effect of GROUP, *F*_(5,83)_ = 180.3, *P* < 0.001; main effect of TIME, *F*_(13,502)_ = 3.385, *P* < 0.001; interaction of GROUP × TIME, *F*_(65,502)_ = 3.315, *P* < 0.001).

A *post hoc* analysis indicated that the MEP amplitudes after stimulation in the 75% RMT-cTBS under both the MMB and K/X groups were significantly decreased compared with those in the no-TBS group (*P* < 0.001). In contrast, the MEP amplitudes were significantly increased in the iTBS groups compared to the no-TBS group (*P* < 0.001, respectively).

MEPs were strongly facilitated immediately after motor cortical cTBS at 80% of the RMT (Figure 2B), lasting up to 25 min under both the MMB and K/X anesthesia (*P* < 0.05; Figure 2B, bottom trace and 4B red, pink line). Similarly, iTBS also facilitated MEPs, lasting up to 25 min under both anesthetic conditions (*P* < 0.05; Figure 4B green and blue line). A two-way ANOVA revealed a significant difference in the normalized MEP amplitude over time (*P* = 0.0003), while *post hoc* comparisons by SPSS (Cary, NC, USA) indicated that the MEP amplitudes were significantly higher than those in no-TBS controls at all time points (asterisks in Figure 4 denote significance).

Although identical stimulus artifacts of 20 ms (50 Hz) were observed, motor responses were not evoked during TBS at any of the stimulus intensities (Figures 2A,B; inside dashed line boxes).

An ANOVA revealed a significant main effect of group on MEP, whereby the effects of the stimulation differed among the six groups (main effect of GROUP, *F*_(5,83)_ = 127.6, *P* < 0.001; main effect of TIME, *F*_(13,502)_ = 22.273, *P* < 0.001; interaction of GROUP × TIME, *F*_(65,502)_ = 1.642, *P* < 0.001).

A *post hoc* analysis indicated significant increases, compared to the no-TBS group, in the MEP amplitudes after the stimulation in both the 80% RMT-cTBS and RMT-iTBS for the MMB and K/X groups (*P* < 0.001).

Multiple comparisons between the 80% RMT-cTBS/MMB groups and the no-TBS group were conducted at each time point. Our results indicated the MEP amplitudes in the 80% RMT-cTBS group to be significantly increased compared with those in the no-TBS group at several time points (20, 22, and 25 min following stimulation: *P* = 0.004, 0.01, and < 0.001, respectively). Differences in the increase of MEP amplitudes were observed immediately following stimulation and persisted for more than an hour (data not shown), suggesting persistent cTBS effects on the MEP amplitudes.

**Figure 5:**
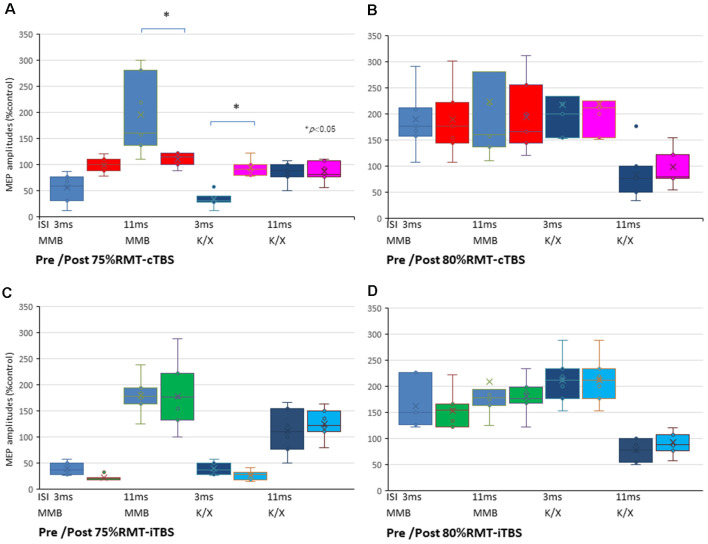
SICI and ICF changes after cTBS and iTBS at different stimulus intensities. **(A)** A multiple comparisons test revealed that the SICI at an ISI of 3 ms was significantly suppressed only after 75% RMT-cTBS under the K/X anesthesia (*P* = 0.046, < 0.05). In contrast, the ICF at an ISI of 11 ms was significantly suppressed only after 75% RMT-cTBS under the MMB anesthesia (*P* < 0.001), while the SICI at an ISI of 3 ms under such anesthesia tended to be suppressed, however, the result was not statistically significant. **(B–D)** Finally, the 75% RMT-iTBS, 80% RMT-cTBS, and 80% RMT-iTBS did not have any effect on either the SICI at an ISI of 3 ms or the ICF at an ISI of 11 ms. Colors in the graph represent each condition pre- and post-TBS (red: cTBS/MMB, pink: cTBS/K/X; green: iTBS/MMB; light blue: iTBS/K/X at 75% and 80% of the RMT, respectively) and anesthesia (blue: MMB; dark blue: K/X, respectively). *P < 0.05.

The SICI at an ISI of 3 ms was significantly suppressed only after 75% RMT-cTBS under the K/X anesthesia (*P* < 0.05). In contrast, the ICF at an ISI of 11 ms was significantly suppressed only after 75% RMT-cTBS under the MMB anesthesia (*P* < 0.001), while the SICI at an ISI of 3 ms under such anesthesia tended to be suppressed, however, the result was not statistically significant (Figure 5A). Finally, the 75% RMT-iTBS, 80% RMT-cTBS, and 80% RMT-iTBS did not affect either the SICI at an ISI of 3 ms or the ICF at an ISI of 11 ms (Figures 5B–D).

## Discussion

Two main findings were reported in the present study. First, MEPs following paired electrical motor cortical stimulation showed inhibition (ISI of 3 ms) and facilitation (ISIs of 11 ms) patterns under MMB anesthesia. In contrast, the K/X anesthesia completely blocked facilitation at an ISI of 11 ms, while inhibition at an ISI of 1 ms and facilitation at an ISI of 3 ms were preserved. This phenomenon is reminiscent of the SICI and ICF in the human motor cortex observed through paired-pulse TMS. Our data indicate that ICF in the rat motor cortex is glutamate-mediated. Second, cTBS, as well as iTBS, strongly facilitated MEPs stimulus intensity for up to 25 min under both the MMB and K/X anesthesia conditions.

Differences in MEPs evoked by TMS have also been documented for different pulse shapes (monophasic vs. biphasic) and different orientations of the electric field (Nakamura et al., 2016; Shirota et al., 2017). While current density close to the electrodes is higher than that in between electrodes, it is more uniform with TMS. Stimulation focus with biphasic stimulation remains unclear (whether at the anodal or cathodal, or in between the electrodes). Preliminary direct comparison studies between epidural bipolar-biphasic-triple pulse and direct cortical monopolar stimulation resulted in similar MEPs. Advantages and limitations of the epidural cortical stimulation have been previously discussed by Kosugi et al. (2018). Given that epidural stimulation is minimally invasive and that it activates pyramidal neurons trans-synaptically *via* cortical interneuron activation, we used epidural bipolar-biphasic-triple pulse for our MEP recordings (Mishra et al., 2017).

### MEP Basic Waveforms

As reported by Mishra et al. (2017), we used a train of three pulses (3-ms interval short train biphasic) to enable the selective activation and temporal summation in the motor cortex (Amassian et al., 1990). This configuration is important as a single pulse causes the activation of both the motor cortex and subcortical structures. Therefore, the MEP latencies of all our results (14.1 ± 0.88 ms in latency, not including waveforms with latencies < 5 ms) imply the activation of rat corticospinal descending motor pathways (Mishra et al., 2017). Indeed, we previously demonstrated that MEPs elicited by direct cortical stimulation (overall conduction velocity of approximately 18 m/s) disappeared after the transection of the corticospinal tract (Kamida et al., 1998). Furthermore, we confirmed that, although single-pulse stimulation also evoked MEPs, it did not activate those of interest for us, which had a higher threshold (approximately three times) and exhibited shorter (approximately 5 ms) latencies (data not shown). It should be noted that electrical stimulation of the corticospinal tract elicits excitatory post-synaptic potentials (EPSPs) in forelimb motoneurons, which are mediated by multi-synaptic excitatory corticofugal pathways and not exclusively by corticospinal axons (Alstermark et al., 2004). Indeed, a localized lesion of the rat corticospinal tract did not affect the size of the short-latency MEPs by TMS over the motor cortex, while mixed descending inputs contributed to the long latency MEPs (Nielsen et al., 2007). Also, the contribution of corticospinal axons and other descending pathways for MEPs production remains unclear (Oudega and Perez, 2012). Similarly to urethane, i.e., a compound commonly used for synaptic plasticity studies (Reynolds et al., 2001; Sykes et al., 2016), we here confirmed that MMB anesthesia (applicable for survival experiments) was also favorable for multi-synaptic corticospinal MEPs and provided continuous stable conditions for MEP recordings.

### SICI and ICF in the Rat Motor Cortex With an Electrical Train of Three Pulses

Contrary to reports of healthy human controls with paired-pulse TMS, a CS at 80% of the RMT preceding paired electrical motor cortical stimulation-induced inhibition (ISI of 1 ms) and facilitation (ISIs of 3 and 11 ms) patterns under the MMB anesthesia. In contrast, a CS at 75% of the RMT preceding TS-MEPs showed inhibition (ISI, 3 ms) and facilitation (ISI, 11 ms) patterns comparable with human results (Kujirai et al., 1993; Rothwell, 1997).

These results were obtained under anesthetic conditions, i.e., GABA-A agonist, midazolam-based MMB, and NMDA antagonist ketamine-based K/X. Inhibitions at an ISI of 1–2 ms were comparable between the anesthetics. Indeed, inhibitions at an ISI of 1–2 ms have been considered to include an axonal refractory period that is not mediated by GABA-A interneurons (Kujirai et al., 1993; Rothwell, 1997). Similarly, an ISI of 3 ms, which is presumed to be a GABA-A-mediated inhibitory phase in healthy humans, was facilitated and comparable between both anesthetics. Each CS-TS train consisting of three pulses (3-ms interval short train, biphasic) should be considered as both trains overlap, and each pulse may interfere at an ISI shorter than 6 ms. This differs fundamentally from the paired single pulse CS-TS TMS paradigm. Specifically, the assessment of SICI at an ISI of 3 ms with certain conditioning intensities can be contaminated by facilitatory effects, such as short ICF (Peurala et al., 2008). Given that both the CS intensities (75 and 80% of the RMT) are constantly subthreshold during TBS (see Figure 2; 5 μV of amplitude calibration in the fourth trace, inside dashed line-boxes), we suggest minimal intensity contamination by intracortical facilitatory influences at this level of conditioning.

Facilitation at an ISI of 11 ms, which is considered to be a glutamate-mediated facilitatory period (Ziemann et al., 2015), was completely blocked under NMDA antagonist ketamine-based K/X anesthesia. This result is consistent with a previous hypothesis proposing that ICF (facilitation at an ISI of 10–15 ms in humans) strongly correlates with excitatory glutamatergic interneurons within the motor cortex depending on NMDA receptor activation (Ziemann et al., 2015).

### cTBS of the Rat Motor Cortex Intensity Dependently Facilitates MEP

Our results demonstrate that 75% RMT-cTBS inhibits while iTBS enhances the neuronal activity and that both 80%-cTBS and iTBS enhance neuronal activity in the cerebral cortex. There are several possible interpretations of our results, indicating that cTBS strongly facilitated MEPs in a stimulus intensity-dependent manner under both the MMB and K/X anesthetic conditions.

Our finding contrasts previous results suggesting that memantine, i.e., a non-competitive NMDA receptor antagonist, blocked both the suppressive effects of cTBS and the facilitatory effects of iTBS. Similarly, it contrasts findings showing that D-cycloserine, i.e., a partial agonist at the NMDA receptor glycine-B biding site, switched the after-effects of iTBS facilitation to inhibition in the human motor cortex (Huang et al., 2007; Teo et al., 2007). Indeed, Hsieh et al. (2015) reported iTBS-MEP facilitation and cTBS-MEP inhibition under xylazine and tiletamine-zolazepam (including tiletamine, a compound that is chemically related to ketamine and fundamentally employs the same mechanisms) anesthesia in rats. A combination of ketamine (90 mg/kg) and xylazine (10 mg/kg) is frequently used for facilitation after paired stimulation to preserve motor responses (Mishra et al., 2017). Anesthetic combinations must be carefully chosen in animal studies concerning stability, MEP preservations, “pseudo potentiation,” non-survival experiments (urethane), and enhancement of GABA transmission (midazolam) or NMDA blockade (e.g., ketamine; Sykes et al., 2016). Strikingly, the present study demonstrated that MEPs were facilitated by either continuous or intermittent TBS under effective doses of anesthetics, including the GABA-A agonist, midazolam, and the NMDA antagonist, ketamine, in the rat motor cortex.

Motor cortical conditions of the subjects, including inter-individual variability (Hamada et al., 2013), and stimulus parameters, such as current direction (Shirota et al., 2017), intensity, and duration of cTBS, alter suppressive or facilitative MEP amplitudes.

An RMT stimulus intensity of 80% in the present experimental settings for anesthetized rats may exceed that of the 80% AMT in the awake human motor cortex. Indeed, cTBS increased motor cortical excitability with a relatively higher 80% AMT intensity, while it was depressed with a lower intensity. The optimal stimulus intensity was not 80% of AMT in every subject (Sasaki et al., 2018).

Similarly, low-intensity, short-interval (300 pulses) cTBS was found to depend on the intensity and to facilitate MEPs at 70% of the RMT and inhibit them at 65% of the RMT, without significant effects on the SICI (Doeltgen and Ridding, 2011). The authors speculated the 70% RMT-cTBS300 to provide sufficient stimulation to breach the activation threshold of intracortical facilitatory interneurons. In contrast, the 65% RMT-cTBS300 was suggested to both facilitate intracortical inhibitory influences and inhibit intracortical facilitatory influences on corticospinal neurons. Lower activation thresholds for intracortical inhibitory interneurons, compared to facilitatory interneurons, exist within a few percent of stimulus intensities (Kujirai et al., 1993).

*In vitro*, low-intensity magnetic stimulation hyperpolarizes action potential thresholds, and increases evoked spike frequency without altering the resting membrane potentials and input resistance (Tang et al., 2016a).

An epidural corticospinal MEP study revealed different intracortical facilitatory and inhibitory neuronal origins that while the 80% AMT-iTBS leads to a rapid increase in the excitability of the cortical mechanism that generates later I-waves, the cTBS preferentially affects the amplitude of the I1 wave (Di Lazzaro et al., 2008). Furthermore, TBS protocols have also been conducted for a longer time compared to reversed facilitatory and inhibitory effects (Gamboa et al., 2010).

The lack of low-intensity 75% RMT-cTBS on the SICI is consistent with previous results (Doeltgen and Ridding, 2011). Considering that the animals in the present study were under anesthetic conditions (GABA-A agonist or NMDA antagonist), the inhibitory effects on the SICI and ICF in response to cTBS may have been affected, as well as the facilitatory effects on the SICI in response to iTBS.

Ketamine, i.e., an NMDA receptor antagonist that indirectly facilitates glutamate neurotransmission through the AMPA receptor, decreased MT based on the administered dose and was shown to enhance MEP response to TMS (Di Lazzaro et al., 2003).

The effect of ketamine possibly suggests an additional contribution of fast ionotropic glutamatergic neurotransmission, most likely at the glutamatergic synapses of these axons onto corticospinal neurons (Ziemann et al., 2015).

The fact that MEPs were facilitated after cTBS or iTBS under ketamine anesthesia, while a lack of ICF was observed, may indicate that AMPA and NMDA transmission are differently involved in TBS effects and paired-pulse CS preceding TS-MEPs.

To understand the underlying mechanisms and to verify their compatibility with human results, further experiments with altered combinations of these cTBS parameters are warranted.

### Limitations and Future Work

A limitation of the present study may lie in the utilization of focal short-burst triple-pulses for stable selective activation and temporal summation in the motor cortex for both CS and TS, as it represents a fundamentally different paradigm to that of the paired single pulse CS-TS TMS. Indeed, the single pulse CS-TS paradigm, even with a high threshold, should be confirmed as a strict benchmark for TMS studies. A stimulus strength of 80% of the RMT for CS and TBS, which might exceed that of 80% AMT, could be reduced in future studies.

Although an ideal-smaller-size, non-invasive animal TMS-coil design for equivalent spatial resolution has been proposed by Tang et al. (2016b), the stereotactic frame under anesthesia conditions is required. Low-intensity electrical iTBS applied to the contralesional hemisphere enhanced functional recovery even at the subacute stage after stroke (Boddington et al., 2020). Effective neuromodulation for symptomatic animal models connected to the stimulator requires repeated sessions under anesthetic drug-free, freely-moving, awake conditions. A reliable, minimally invasive, and quantitative motor mapping and MEP recording method in anesthesia-free conditions are warranted for elucidating the mechanisms underlying cortical motor reorganization. Establishing stable and reproducible conditions for RMT, AMT, and MEPs for long-term evaluations in awake, freely-moving rodents is also necessary (Kosugi et al., 2018). Furthermore, Hoogendam et al. (2010) presented—in a critical review—seven lines of evidence suggesting that neuromodulation of the rTMS is a result of the induction of synaptic changes resembling long-term potentiation and depression (LTP and LTD). Evidence includes similarities in stimulation temporal patterns required for induction, duration of changes, and sensitivity to pharmacological interventions. This is consistent with the hypothesis that motor cortex stimulation can activate MEPs, as well as cellular and molecular mechanisms underlying different forms of synaptic plasticity, such as LTP and LTD, for future neuromodulation-based therapeutic strategy (Müller-Dahlhaus and Vlachos, 2013; Rodger and Sherrard, 2015).

## Conclusions

Paired-stimulation corticospinal MEPs induced inhibition and facilitation patterns that were similar, but not identical, to those of the SICI and ICF in the human motor cortex obtained when using paired-pulse TMS. Both continuous and intermittent TBS-induced MEP facilitation under two anesthetic conditions. Continuous TBS parameters in the rat motor cortex should be further explored to elucidate the underlying mechanisms.

## Data Availability Statement

The raw data supporting the conclusions of this article will be made available by the authors, without undue reservation.

## Ethics Statement

The animal study was reviewed and approved by Ethical Committee of the School of Medicine, Oita University.

## Author Contributions

MF and YK designed the research paradigm. MF, HF, and YK performed the research investigations. MF and HF analyzed the data and wrote the article. All authors contributed to the article and approved the submitted version.

## Conflict of Interest

The authors declare that the research was conducted in the absence of any commercial or financial relationships that could be construed as a potential conflict of interest.
